# Guarana

**Published:** 1874-04

**Authors:** 


### Guarana.

Attention has recently been called to the therapeutical value of a comparatively new remedy called Guarana. The cases in which it is claimed to be of value are those in which there exists some derangement of the stomach and bowels. It has been tried in sick headache, diarrhæa, and dysentry, and other affections of a similar nature, and marked improvement is reported to have attended its administration.

Guarana has been used for some time by South American physicians, and also to a limited extent in Europe. It is prepared from the dried resinous juice and seeds of the Paullinia Sorbilis, and in France, has been used under the name of Paullinia. Mr. Peabody informs us that he has obtained a quantity of this remedy, and physicians who desire to test its qualities, can obtain the pure article.

